# Measuring Psychological Change and Predicting Recidivism Following the Swedish One-to-One Program

**DOI:** 10.3389/fpsyt.2019.00811

**Published:** 2019-11-29

**Authors:** Anne H. Berman, Mikael Gajecki, Per Morien, Philip Priestley

**Affiliations:** ^1^Center for Psychiatry Research, Department of Clinical Neuroscience, Karolinska Institutet, Stockholm, Sweden; ^2^Stockholm Health Care Services, Stockholm County Council, Stockholm, Sweden; ^3^Stockholm Center for Dependency Disorders, Stockholm, Sweden; ^4^Department of Psychology, Örebro University, Örebro, Sweden; ^5^Retired, Bristol, United Kingdom

**Keywords:** recidivism, individual psychological intervention, psychological tests, prison, probation, criminal justice, naturalistic study

## Abstract

The One-to-One program aims to reduce criminal recidivism among prisoners shortly awaiting release, and among probationers. Of 1,484 program participants in Sweden, 776 contained adequate data for analysis. Pre- and post-program scores were available for the Alternative Thinking Test, Levenson's Locus of Control Scale, Skill Survey, Citizen Scale, and Problem Checklist, all areas addressed in the program. This study examined predictive properties of test scores and background characteristics regarding recidivism, as well as differences between sub-groups. All post-tests indicated pro-social changes. Older participants were more likely to complete the program. The most potent predictor for non-recidivism was program completion, with non-completers 64% more likely to re-offend. Significant associations occurred between recidivism and the tests measuring skill improvement over time, chance locus of control pre- and post-program, and attitudes and values (Citizen Scale), partly supporting the theory behind the program.

## Introduction

Criminal justice authorities have long been struggling to find effective ways to reduce recidivism in crime. The conviction that rehabilitation of individuals in the criminal justice system would not work was widespread following (1) negative evaluation of treatment programs from the 50s and 60s, leading to use of the term "nothing works." A slow return to belief in the value of rehabilitation came in the 1990s, when meta-analyses showed that some programs did work, particularly when they followed principles adapting content to participants' *risk* for reoffending, their specific offending-related problems or *needs*, and were built on *responsivity* to participants' learning styles (2). This development led to what amounted to a movement among policy makers and criminal justice staff, leading to intensive activity towards identifying "what works" and developing and implementing programs that would work. Among treatment programs that early on were shown to be effective in reducing criminal recidivism, 75% are based on cognitive behavioral theory (CBT) and are multifaceted (3). With a focus on criminogenic needs—cognitive deficits and offending-related attitudes and beliefs (4)—additional effective components include a sound conceptual model, attention to the responsivity principle, role playing/modeling, and social cognitive skills training (5).

The One-to-One (OTO) program was developed by Philip Priestley in Great Britain in 1993 with the aim of satisfying the above criteria for effective programs among probationers. In the OTO-program the focus lies on finding examples from daily life and on giving homework, which gives the client an opportunity to practice actual execution of the problem solving skills (6). The format of this program is "one-to-one"; i.e., a counselor works individually with a client for up to twenty sessions over a period of several months. The OTO program is based on cognitive-behavioral principles and focuses on specific areas. Skills in areas such as interpersonal/social (cognitive) problem-solving, social skills, and self-control are trained. Work is also done in the areas of attitudes and values and thinking (as in cognitive restructuring). The main focus is on problem solving, and criminal behavior is regarded as a problem to be solved. The program integrates the above-mentioned concepts and measures changes in each specific area over time. The OTO program has been accredited for use by community probation services in England and Wales (7) and as a national program for use both in probation and prison settings in Norway (8). It has also been implemented on a small scale in Lithuania (9) and piloted in the Netherlands (10). A small scale study in the English West Mercia probation area reported a 24% reduction for 51 OTO completers in observed reconvictions, compared to those expected based on national statistics (7).

In an effort to ensure the quality of the programs offered, the Swedish Prison and Probation Service has adopted the "what works" initiative and consequently aims to implement only evidence-based programs, with 17 programs currently accredited (11). One of the programs accredited under this initiative is the OTO CBT program for addressing criminal behavior (12). The OTO program was the first program in Swedish criminal justice that was not conducted in a group format. A first evaluation in the Swedish context showed a 25% reduction in reoffending for 350 OTO program completers in prison and probation, compared to 7,280 non-participants, with incomplete participation associated with 28% higher risk of recidivism (13). A more recent Swedish evaluation reported that OTO program completion was associated with a 15% lower risk for reoffending compared to controls, whereas incomplete participation was associated with a 61% increased risk (14). An individual program might be more suitable for some participants, and logistic considerations as well as personal characteristics may be considered when choosing an individual format. Different delivery formats in terms of program length have been used (6), where the format used in Sweden consists of 20 one-hour sessions with the participant.

Most evaluations of offending behavior programs focus on one outcome measure only; i.e., either recidivism as a binomial variable, or psychological change of some kind (15). As the main aim of all these programs is to reduce re-offending, the recidivism view of outcome success is easily understood and adopted. However, it tells us nothing of the link between psychological change and change in recidivism, in terms of what actually happens within the program. As one of the characteristics associated with program success is that the program is based on a "sound conceptual model" (3), it is also important to see how this conceptual model holds up to scrutiny. This study examines post-program psychological changes as measured by the tests in this program and their associations with the outcome of recidivism, as well as pre-program test-based predictors of recidivism. This becomes an examination of the theoretical basis for this program, and leads to the following aim and research questions.

We aim to explore whether background data or test data have predictive properties regarding completion and recidivism and whether test outcome scores differ significantly between completers/non-completers and recidivists/non-recidivists. The specific research questions were whether a) test scores differed significantly in the entire cohort over time; b) whether completers and non-completers differed in background data and pre-test scores and, where available, whether program completers differed from norm data c) whether recidivists and non-recidivists differed significantly in background data and test scores d) whether non-completion of the OTO program could be predicted by background data and pre-program scores; and e) whether recidivism rates could be predicted by background data, pre- and post-program scores, and/or change scores over time for pre-/post-tests.

## Materials and Methods

### Sample

The data for this study were collected by the Swedish Prison and Probation Service. The original data set consisted of 1,484 participants who entered the program between 2000 and 2008. The program was formally accredited in 2003 and by 2008 it was offered in 27 of 60 probation units in Sweden and 11 of 55 prisons (13). Clients with a medium to high risk for recidivism were considered appropriate for the program. Prior to recruitment, an evaluation interview took place to assess motivation and suitability, and both client and interviewer discussed whether they thought the client would benefit from the program and if so, made a joint decision for the client to enter the program (H. Nyberg, personal communication, March 24, 2009).

**Figure 1:**
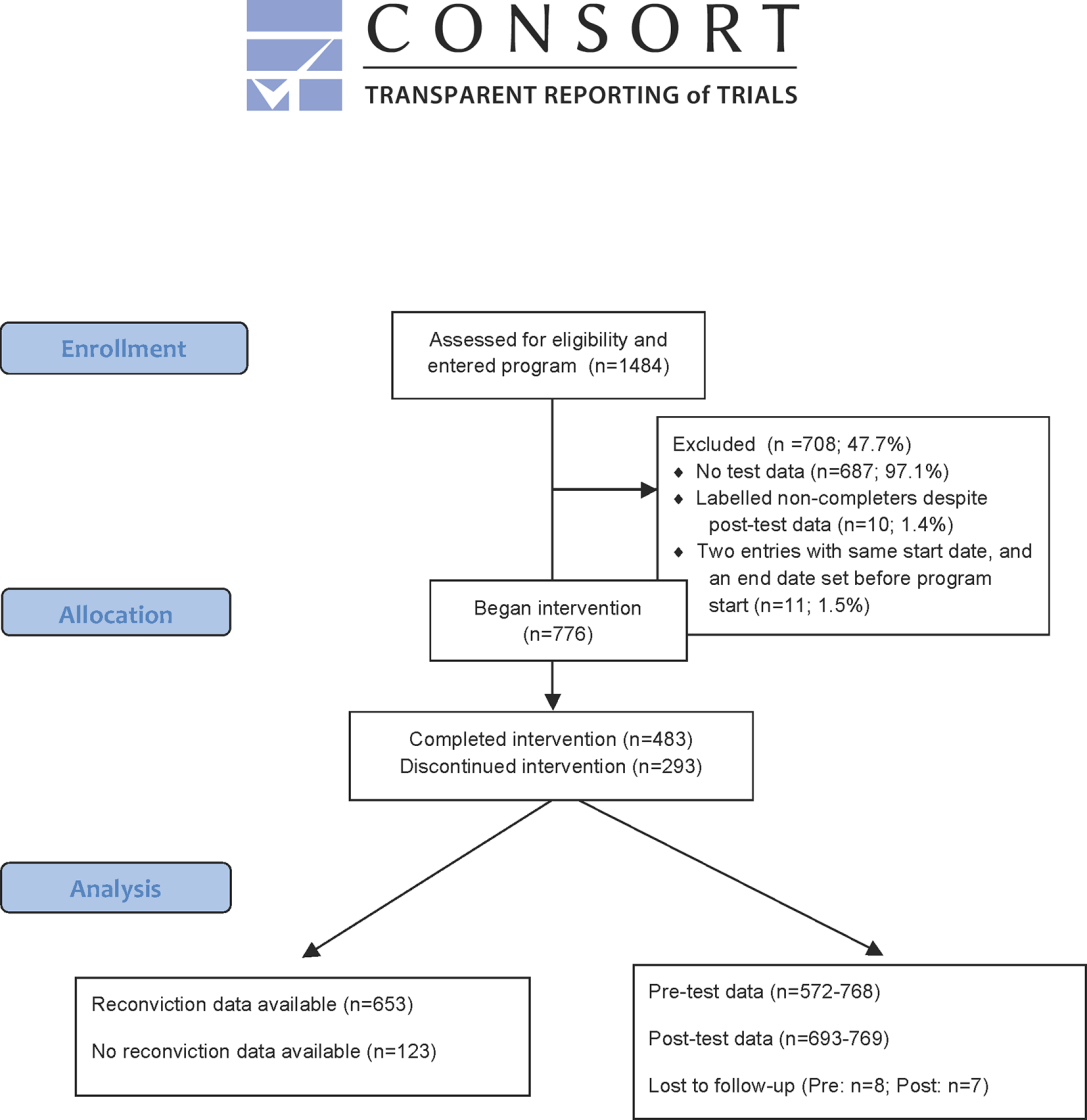
Participants in the Swedish One-to-One program (CONSORT 2010 Flow Diagram).

Of the 1,484 cases included in the data set, 687 lacked pre- or post-test data and were excluded. Due to inconsistencies in the data, such as dates, number of sessions, completer/non-completer status, and multiple registrations a further 21 cases were excluded. The final data set consisted of 776 cases (see **Figure 1**).

Participant characteristics were available regarding age, gender, program completion, and recidivism up to 5 years following program participation. When starting the program participants were 27.92 years old on average [standard deviation (sd) = 9.33]. Almost all participants were male (92.3%). Of the 776 participants, 483 were completers and 293 were non-completers (see **Table 1**). All participants were convicted individuals who had access to the program during their term in probation, prison, or electronic monitoring. Data on recidivism were obtained from Swedish Prison and Probation Service records from September 1, 2007 or after a maximum of 5 years following program completion. The mean follow-up time was 854.51 days (sd = 673.42; range 0–1,827 days).

**Table 1:** Sample characteristics for One-to-One program participants.

	N	n	%	Min.	Max.	Mean	Std. Dev.
*Background characteristics*							
Age at program start	771			15	66	27.92	9.33
Male	776	716	92.3%				
Female	776	60	7.7%				
*Program participation* No. of sessions	686			1	25	13.41	7.47
Completers	776	483	62.2%				
Non-completers	776	293	37.8%				
*Pre-/post criminality* No. of previous convictions	314			1	8	2.45	1.62
Recidivist	653	215	32.9%				
Non recidivist	653	438	67.1%				

### Outcome Measures

The primary outcome measure was recidivism, defined as relapse into criminal behavior, which had been noted in the criminal justice system in terms of trial and reconviction during the follow-up period after the intervention. The terms recidivism, reconviction, and reoffense are used interchangeably.

Secondary outcome measures are scores on five different tests used within the OTO program as a basis for counselor-participant discussions in the sessions. No psychometric data were available from Swedish samples on these tests, but they were retained to facilitate international comparability. **Table 2** shows the measures used in the study with information on number of items, the construct measured, and references.

**Table 2:** Measures used in the study.

Measure	Items	Construct	References
Alternative Thinking Test (ATT)	4	Capacity to solve problems	Spivack, et al., (16)
Levenson's Locus of Control Scale (LLOCS)	24 (3 subscales)	Multi-dimensional locus of control. Subscales Internal, Powerful Others, and Chance	Levenson (17)
Skill Survey	20	Social skills. Questions taken from Goldstein's Prepare Curriculum	Developed originally by Goldstein (18), 20 questions were selected for OTO program by Priestley (6)
Citizen Scale	4	Attitudes and values	Developed originally by Schneider (19), the Citizen Scale subscale was selected for OTO by Priestley (6)
Problem Checklist	110 (11 subscales)	A questionnaire regarding problems in different areas of life	Constructed by James McGuire and adapted for the Swedish OTO by the Swedish Prison and Probation Service in Priestley (6)

### Measures

#### Alternative Thinking Test

The Alternative Thinking Test (ATT) was developed as a means to measure the ability to generate solutions to problems, the *optional thinking* skill as defined by 16 The test consists of four different problems to which the test person generates as many solutions as possible. The number of alternative solutions is the test score. The test scores range from 0 with no upper limit, though scores on each item rarely exceed 12. This is the only test in OTO aiming to directly measure a skill rather than collecting participants' self-assessments. The ATT has been found to negatively correlate with self-harm in deliberate self-harm patients with no previous history of self-harm (20), verbal fluency, and attention in schizophrenic patients (21), and being a psychiatric patient (22). Pilot studies in Somerset and Cardiff have shown a significant increase in scores for OTO participants (6).

#### Levenson Locus of Control Scale

The Levenson Locus of Control Scale (LLOCS) is a well-researched test for measuring participants' views of their locus of control. The LLOCS scale consists of 24 items scored from -3 to +3, with 8 items in three subscales: Internal, Powerful Others, and Chance (IPC). The test is scored by adding 24 points to each subscale, yielding scores ranging from 0 to 48 for each subscale. As these scores are mutually independent, any combination of scores is possible. Norms based on an undergraduate U.S. student sample (n=161) with a mean age of 23.1 years yielded mean (sd) scores of 34.4 (5.4) on the Internal subscale, 24.4 (6.8) on the Powerful Others subscale, and 24.8 (6.1) on the Chance subscale (23). Conceptually, factor analysis has shown that the three dimensions of the LLOCS constitute different factors and the construct validity is satisfactory (24). Further support for the three-factor model emerged from a confirmatory factor analysis (25), and an analysis of three scales by Lindbloom & Faw (26). A pilot study found no significant changes over time in any of the subscales in pre- and post-program LLOCS scores for OTO participants (6); the test was therefore excluded in later versions of the program, after 2008.

#### Skill Survey

The Skill Survey measures strengths and weaknesses in the participant's social skill set. The survey consists of 20 items, selected from 50 items presented in the Prepare Curriculum (18). Each item requires the participant to rate how well they use a certain skill, scored from 1 (not good) to 5 (very good), resulting in a score ranging from 20 to 100. In the OTO program, the survey is used to reflect changes in self-reported social skills levels. In pilot studies of OTO significant increases in skill levels have been reported for participants (6).

#### Citizen Scale

The Citizen Scale is a subset of a larger test designed by Anne Schneider (19), used in the OTO program under the name "The kind of person I am" to monitor changes in participants' views of themselves as being "good" citizens during the program. The test consists of four items, scored from 1 to 7 where each extreme represents the opposite of the other, for example "breaks rules" in contrast to "obeys rules"; the total score generated ranges between 4 and 28. Within the OTO program, the Citizen Scale score has demonstrated predictive power on the rate of reconviction, with higher scores linked to a lower reconviction rate (12).

#### Problem Checklist

The Problem Checklist (PCL) consists of 110 items used to assess the presence of criminogenic problems in the participant's life and divided into 11 subscales which reflect specific problem areas: work and unemployment, accommodation, money and financial pressures, alcohol, substance use, gambling, physical and mental health, social relationships, peer group pressure, family relationships, and family offending. Participants score each item from 0 to 10, where 0 means that the problem described in the item is not a problem for the participant, and 10 means that the problem is very serious; total scores including all subscales range from 0 to 110. The list was developed for the OTO program based on items generated by probation officers in Kent and thereafter modified and translated by the Swedish Prison and Probation Service. Within the program, the checklist is delivered in three parts during the first three sessions of the assessment part of the program. The checklist is also given in its entirety during session 19 as a post-test to see if there is any reduction in problem perception. Feedback regarding the division into subscales is revealed to the participant after the last module has been delivered. Within the OTO program, a pilot study showed that lower PCL scores at pre-test were associated with lower reconviction rates, and a later study showed significant positive post-program changes over time for all subscales on perceived problems (6).

### The One-To-One Program

The structure of the One-To-One program is briefly outlined in **Table 3**. Each session follows a common structure throughout the program. Initially, any pressing issues that need to be handled are dealt with, followed by a review of homework tasks from the previous session. After this, the focus is on session-specific content, and the session ends with action plans for the time until the next session. Relapse prevention issues are continually addressed through discussion of the possibility of setbacks and of whether they really indicate failure.

**Table 3:** One-to-One program content.

Module	Sessions	Significant aspects
Pre-program motivational session	Pre-prog.	The counselor and the participant get to know each other. The participant is introduced to the program, and the program leader elicits statements about the participant's motivation for joining the program and reinforces them. Practice exercises are introduced.
Assessment	1–5	Assessment tests are completed by the participant. The counselor conducts a behavioral analysis focusing on antecedents, behavior, and consequences of the participant's offending behavior, and also proposes a personalized theory of the participant's offending.
Skills training	6–13	The participant works with applied training on problem solving, social skills, cognitive restructuring, moral training, goal setting, and self-management.
Application	14–20	Problem solving and social skills training are further practiced. The counselor also chooses areas from the skills training part deemed beneficial for the participant for further practice. This part ends with completion of post-tests, after which the participant formally exits the program.

### Ethical Considerations

Written informed consent was given by the participants for the use of data for research purposes, according to a routine established by the Swedish Prison and Probation Service. Data were provided by agreement between the Service and author PP, in a file where personal identifiers such as civic registration number and name had been removed. For research purposes, data such as age, gender, time of attending the program, and classification of the offense were retained in the data file.

### Statistical Analyses

The statistical analyses were performed using statistical package SPSS 16.0 for Mac. For baseline comparisons between included and excluded participants, chi-square tests were used for non-parametric data and independent samples t-tests for parametric data. For comparisons between pre- and post-program scores, paired samples t-tests were used, as well as within-group effect sizes according to Cohen's *d* (27). For comparisons of pre-program scores between program completers and non-completers, and comparisons of pre- and post-program scores between recidivists and non-recidivists, independent samples t-tests were used. In order to control for the effects of mass significance, increasing the probability of type I errors, a Bonferroni-corrected probability of p = 0.05/76 = 0.0007 was used. According to Lazerle & Mulaik (28), this is an unnecessarily conservative measure that may increase the risk for type II errors (false negatives). Therefore, in this study, results are reported according to a multi-stage method suggested by Lazerle & Mulaik (28), where the p-value was corrected to (0.05/[76-27] = 0.001), where 76 refers to the total number of significance tests carried out and the 27 refers to the number of significant results according to the first Bonferroni correction (p-value 0.0007).To assess predictive factors for program completion and recidivism, respectively, logistic regression was used.

### Missing Data

Complete data for 776 cases were available for gender, and data for number of previous convictions were available for 314 cases (missing for 462). For pre-program tests, data for 5–204 cases were missing and for post-program tests 7–83 cases were missing (see **Figure 1**). Since data were collected by the Prison and Probation Service, the reasons for missing data are unknown. In the case of the LLOCS pre-test, the difference in missing values between this test and for example the PCL may mainly be due to the fact that the later version of the manual does not include the LLOCS. The large number of missing values in the number of previous convictions variable may be due to the possibility that some of these missing values were intended to mean zero previous convictions and were not actually missing values. No imputation procedures were applied.

### Comparisons Between Included and Excluded Participants

Comparisons between the 687 excluded cases and the 776 cases that remained available for further analysis (see **Figure 1**) were made for gender, age, recidivism, and number of sessions, showing no significant differences (gender, χ2 = 2.29, df = 1, p = 0.130; age, t = -0.645, df = 1,324, p = 0.159; recidivism χ2 = 1.558, df = 1, p = 0.212). However, exclusion from analysis within this study was significantly related to the number of pre-program sessions, with the excluded group having fewer sessions (t = -9.190, df = 1,232, p < 0.001). Data were missing in the excluded group for 13 participants regarding gender, 129 for age, 3 for recidivism, and 158 for number of sessions; in the included group data were missing for 26 regarding gender, 123 regarding recidivism, and 89 regarding number of sessions.

## Results

### Paired t-Tests Comparing Pre- and Post-Test Data

All paired t-tests comparing pre- and post-program scores over time yielded significant differences, using the multistage Bonferroni corrected p-value of 0.001 (**Table 4**).

**Table 4:** Comparisons over time of results on pre- and post-program tests

Measure (pre-; post-n)	Pre-program mean (sd)	Post-program mean (sd)	t	Degrees of freedom (df)	p	Pre-/post-program difference (sd)	Cohen's d
**Alternative Thinking Test** (n = 760; n = 758)	12.27 (4.36)	15.23 (5.78)	-12.20	464	<0.001	–3.06 (5.40)	0.58
**LLOCS** (n = 572; n = 693)							
Internal	37.17 (4.76)	38.41 (4.61)	-5.19	396	<0.001	–1.24 (4.78)	0.26
Powerful Others	22.86 (7.11)	21.50 (6.99)	4.13	396	<0.001	1.36 (6.58)	0.19
Chance	25.12 (6.51)	22.72 (7.36)	7.91	396	<0.001	2.40 (6.06)	0.35
**Skill Survey** (n = 729; n = 767)	69.22 (12.00)	75.19 (12.38)	-11.51	471	<0.001	-5.97 (11.27)	0.49
**Citizen Scale** (n = 768; n = 768)	19.76 (4.02)	22.27 (3.88)	-14.02	474	<0.001	-2.50 (3.89)	0.64
**Problem checklist (PCL)** **^a^**							
(n = 765; n = 769)							
Work and unemployment	29.11 (18.72)	20.29 (17.30)	12.41	470	<0.001	8.82 (15,29)	0.49
Money and financial pressures	26.76 (20.28)	18.20 (18.50)	12.07	470	<0.001	8.56 (15.39)	0.44
Alcohol	14.54 (16.18)	9.26 (12.98)	9.50	470	<0.001	5.28 (12.06)	0.36
Substance use	11.96 (16.84)	6.18 (12.83)	10.18	470	<0.001	5.78 (12.32)	0.39
Gambling	5.52 (13.42)	3.23 (9.44)	5.55	470	<0.001	2.29 (8.95)	0.20
Physical and mental health	19,96 (16.31)	11,56 (12.84)	13.74	470	<0.001	8.40 (13.27)	0.57
Social relationships	16,02 (14.40)	9,38 (11.31)	12.70	470	<0.001	6.64 (11.35)	0.51
Peer group pressure	21.05 (15.56)	12.39 (12.27)	14.78	470	<0.001	8.66 (12.72)	0.61
Family relationships	14.55 (14.24)	9.01 (12.04)	10.69	470	<0.001	5.54 (11.24)	0.42
Family offending	5.34 (8.21)	2.63 (7.33)	5.92	470	<0.001	1.70 (6.25)	0.35

a In the Swedish version of the PCL the subscale on accommodation was exchanged for a subscale on violence that was considered more relevant to the Swedish setting. However, the data available included both PCL versions, although not identified as to which version was used, so results on the accommodation or violence subscales could not presented.

### Completers Compared to Dropouts

Only age at program end showed a significant difference between program completers and dropouts (t = -3.76, df = 748, p < 0.001), where program completers were on average 2.96 years older than non-completers.

### Recidivist Completers Compared to Non-Recidivist Completers

Comparisons of pre- and post-program scores for program completers who were later reconvicted (recidivists) with non-recidivist program completers showed significant differences for the Levenson Chance subscale and the Citizen Scale pre- and post-program tests and for number of previous convictions. Recidivists scored higher on the LLOCS Chance subscale pre- and post-program tests and lower on both pre- and post-program tests for the Citizen Scale. Recidivists also had a significantly higher number of previous convictions (see **Table 5**).

**Table 5:** Significant differences in pre- and post-program means between recidivists and non-recidivists.

Test	Pre-/post-program tests	Recidivist Mean (SD)	Non-recidivist Mean (SD)	t	df	Sig. (two-tailed)
LLOCS Chance	Pre-program	23.50 (7.09)	22.05 (7.00)	–3.95	488	.000
	Post-program	22.96 (6.98)	20.63 (6.89)	–3.43	366	.001
Citizen Scale	Pre-program	18.77 (4.25)	19.95 (4.05)	3.43	647	.001
	Post-program	15.56 (6.04)	15.08 (5.74)	3.50	441	.001
Number of previous convictions	Pre-program	3.26 (1.88)	2.11 (1.36)	–3.62	61.64	.000

### Completers' Locus of Control Compared to Student Sample

Comparison of pre- and post-program scores on the three LLOCS sub-scales for program completers with cross-sectional student test means (23) showed that completers differed significantly on all scores except for the Chance pre-program test. Before and after the program, program completers had higher scores than students on the Internal sub-scale as well as lower scores on the Powerful Others sub-scale; after the program, completers had lower scores on the Chance sub-scale (see **Table 6**).

**Table 6:** Comparison of program completers' locus of control to norm data.

Test	Pre-/Post-test	Student test mean (SD)	OTO test mean (SD)	*t*-value	df	Sig. (two-tailed)
LLOCS Internal	Pre-test	34.4 (5,4)	37,06 (4,83)	13.18	571	.000
	Post-test	“	38,38 (4,66)	17.06	399	.000
LLOCS Powerful Others	Pre-test	24.4 (6,8)	23,01 (7,18)	–4.64	571	.000
	Post-test	“	21,48 (6,98)	–8.37	399	.000
LLOCS Chance	Pre-test	24.8 (6,1)	25,42 (6,76)	2.19	571	.029
	Post-test	“	22,76 (7,36)	–5.54	399	.000

### Predictors of Program Completion and Recidivism

Predictor variables were initially evaluated to identify whether any association existed with the dependent variables (DVs) of program completion and recidivism. For gender, no association was identified, neither for program completion (χ^2 =^ 1.03, df = 1, p = 0.311) nor for recidivism (χ^2^ = 1.38, df = 1, p = 0.279). Gender was therefore not included as a predictor in the logistic regression analyses below. The predictor variables retained were age and all pre-program tests.

For program completion, a total of 554 cases were included in the analysis, and the full model was significant (χ^2^ = 64.94, p < 0.001), accounting for between 11.1 and 15.9% of the variance. Overall, 73.8% of the predictions of completion status were correct, with 95.7 of the completers correctly predicted, but only 18.5% of the dropouts. Only the age variable was significant for predicting non-completion, both age at the beginning of the program (B = 0.40) and at the end (B = 2.57), meaning that for every increase of 1 year in age at the end of the program, the chance for completion increased by a factor of 2.57.

For recidivism among program completers, predictor variables consisted of pre- and post-program scores, the difference between pre- and post-program scores, and the categorical variables of program completion and age as predictor variables. As the predictor variables were as many as 50, a forward conditional analysis was used in order to find the model best predicting the DV. The best-fit model included age at the end of the program, the pre-program LLOCS Chance subscale, the post-program PCL money and financial problems subscale, and the difference over time between pre- and post-program Skill Survey scores. An enter method logistic regression including only these four variables was performed in order to include a maximum of cases with valid data. A total of 365 cases were analyzed and the full model was significant (χ^2^ = 33,73, df = 4, p < 0.0001). This model accounted for between 8.8 and 12.5% of the variance, with 94.5% of non-recidivists successfully predicted but only 17.9% of recidivists accurately predicted. Overall, 71.0% of the predictions were accurate. **Table 7** shows that older completers were less likely to be reconvicted, with an odds ratio (B) of.96; having more post-program financial problems was also associated with being reconvicted, with an odds ratio (B) of 1.02. A larger change over time on the Skill Survey score was also associated with not being reconvicted (B = 0.97).

**Table 7:** Predictors of recidivism among program completers (*n* = 365).

	Variables in the equation	B	S.E.	Wald	df	Sig.	Exp(B)
Step 1	LLOCS Chance pre-test	.03	.02	3.13	1	.077	1.04
Change score Skill Survey	–.03	.01	5.71	1	.017	.97	
Age at end of program	–.04	.01	7.57	1	.006	.96	
Problem Checklist money and financial pressures post-test	.02	.01	11.72	1	.001	1.02	
Constant	–.87	.67	1.67	1	.196	.42	

A second logistic regression analysis was carried out with recidivism as the DV among program completers as well as non-completers (for whom post-program data were lacking) using pre-program data, age and program completion status as predictor variables. A total of 554 cases were analyzed and the full model was significant (χ^2^ = 64.93, df = 18, p < 0.0001). This model accounted for between 11.1 and 15.9% of the variance, with 95.7% of non-recidivists successfully predicted but only 18.5% of recidivists accurately predicted. Overall, 73.8% of the predictions were accurate. The significant variables were: the PCL pre-program money and financial pressures and physical and mental health subscales, the LLOCS pre-program Chance subscale, and completion status^1^. A higher level of perceived financial problems was associated with being reconvicted (odds ratio 1.02), whereas a higher level of perceived health problems was associated with *not* being reconvicted (odds ratio 1.02). Higher scores on the LLOCS Chance subscale were associated with being reconvicted (odds ratio 1.05), and program completers were less than half as likely to be reconvicted (odds ratio 0.36; see **Table 8**).

**Table 8:** Predictors of recidivism among program completers and non-completers (n = 554).

	Predictor variables^a^	B	S.E.	Wald	df	Sig.	**Exp(B)**
Step 1	Alternative Thinking Test	-.01	.02	.06	1	.799	.99
	LLOCS—Internal	.03	.02	.02	1	.144	1.03
	LLOCS—Powerful Others	.01	.02	.02	1	.418	1.01
	**LLOCS—Chance** **^b^**	**.05**	**.02**	**.02**	**1**	**.016**	**1.05**
	Skill Survey	.00	.01	.01	1	.588	1.00
	Citizen Scale	-.04	.03	.03	1	.192	.96
	PCL work and unemployment	-.00	.01	.01	1	.724	1.00
	**PCL money and financial pressures**	**.02**	**.01**	**.01**	**1**	**.004**	**1.02**
	PCL alcohol	-.01	.01	.01	1	.337	.99
	PCL substance use	.00	.01	.01	1	.691	1.00
	PCL gambling	-.01	.01	.01	1	.290	.99
	**PCL physical and mental health**	-**.02**	**.01**	**.01**	**1**	**.040**	**.98**
	PCL social relationships	.02	.01	.01	1	.098	1.02
	PCL peer group pressure	-.00	.01	.01	1	.816	1.00
	PCL family relationships	-.01	.01	.01	1	.228	.99
	PCL family offending	.02	.01	.01	1	.119	1.02
	**Program completion status**	-**1.03**	**.24**	**.24**	**1**	**.000**	**.36**
	Age at program start	.14	.14	.14	1	.290	1.16
	Age at program end	-.17	.14	.14	1	.210	.84
	Constant	-1.42	1.26	1.26	1	.261	.24

a All test variables are from the pre-program testing time point.

b Variables marked in bold were significant predictors of recidivism (not subject to Bonferroni correction).

## Discussion

How to maximize reduction of criminal recidivism has been the main focus of criminal justice activities in Sweden for about 25 years, through the introduction of "what works" concepts and empirical findings (29). Numerous CBT programs have been introduced during this time, and evaluations of recidivism as an outcome have shown that the programs are associated with lower recidivism rates, including the OTO program, with its 15% lower risk of recidivism for program completers, and a much higher risk of 61% for non-completers (14). In this study, our aim has been to elucidate possible associations between psychological changes measured within the OTO program, and recidivism or its absence.

The most important variable protecting against recidivism in OTO-program participants was program completion, with non-completers 64% more likely to re-offend. Numerically, 25.7% of the OTO program completers re-offended compared to 49% of non-completers; the average rate of recidivism for the entire criminal justice population in Sweden varied during this time between about 40% in the year 2000 and declined to about 33% in 2008 (30). Older participants were more likely to complete the program, and they were also less likely to re-offend. Among program completers, all test results improved in a pro-social direction, with most effect sizes in the medium range. Regarding specific test results, *social skills* tended to improve over time among completers who did not re-offend, compared to recidivists. *Locus of control* tests indicated that program completers believed more in internal control and less in chance determination compared to student norms. However, all program participants at baseline perceived a higher internal control over events while ascribing less control to other powerful people compared to student norms. Furthermore, participants at baseline believed in chance as a determinant of life events about equally to norm data. In summary, program completion, being older and improving skills over time seemed to be primary factors associated with non-recidivism, recidivists, whether or not they completed the program, had more prior convictions than non-recidivists. All measures of change over time for program completers indicated positive, pro-social development, with effect sizes varying between 0.19 on Powerful Others locus of control and 0.64 on the Citizen Scale. Nonetheless, aside from a pre-program perception of worse health, only changes in social skills over time were clearly associated with absence of recidivism. Recidivism was associated with perception of greater perceived financial problems and Chance locus of control. We focus our discussion on background and program-related factors found to be associated with recidivism, and aspects of psychological change identified as significant predictors of recidivism or its absence.

Higher age protected from recidivism and was associated with program completion, in line with previous extensive literature (e.g., 31). One possible consequence of this finding could be that an older population should be directed to OTO, whereas a different program, better suited for younger age groups, should be offered them. Recidivists had more previous convictions than non-recidivists, a finding also congruent with previous findings indicating that the number of previous convictions is one of the most important risk factors related to criminality (32, 33). The program completion variable was the most potent in predicting absence of recidivism. In view of the exclusion from analysis of the variable on the number of previous convictions due to missing data, the potency of program completion as a predictor for non-recidivism may have spuriously increased in the logistic regression model. The clear difference in recidivism between completers and non-completers, however, was expected based on prior research.

Notwithstanding these issues, the comparison of OTO participants to matched non-participant controls showed a program effect for reducing recidivism (14) as well as similar findings from other studies comparing CBT program participants to matched controls (e.g., 31) indicating that the program can be ascribed an effect beyond static predictors of recidivism such as age and number of previous convictions. Worth noting, however, is that non-completion of such programs is associated with a higher risk of recidivism compared to matched individuals who did not participate in programs, suggesting that the fact of non-completion can carry a heightened risk (34). Individuals identified as high-risk are more likely to drop out of a program (35). A study of sexual offense cases showed that the largest predictor of dropout from treatment was previous convictions for non-sexual offences. Also, fathers' unemployment is a variable that has shown discriminating properties between completers and non-completers (36). Another study of dropout found that cannabis dependence, criminal history (in terms of previous lifetime arrests), as well as hostility during treatment are factors that independently predict dropout, whereas employment predicts staying in treatment (37). A qualitative study found no difference in motivation to change between completers and non-completers (38), although Wormith & Olver (35) found motivation (not clearly defined) to be a predictor of dropout. A study on differences between dropouts and completers in a court-mandated treatment for spouse abusers found that dropouts were more likely to be younger, not married and more likely to have previous convictions, but also less likely to be depressed when starting the program (39).

In terms of measures of psychological change during the program, improving social skills during the program was found to be a factor protecting from recidivism. The aim of the Skill Survey is to identify strengths and weaknesses in the participant's social skill set, and each of the skills associated with the test items is the focus of modules in the OTO program. An increase in social skills according to the Skill Survey could reflect participant success in utilizing social skills training within OTO, yielding an effect on the targeted outcome of recidivism. The finding that perceived pre-program worse health appeared to protect against recidivism might be interpreted to mean that the criminal lifestyle is challenging and that a person leading that kind of life has to have some measure of good health to persevere in it. Since the PCL physical and mental health subscale does not distinguish between physical and mental it is not clear, however, what this finding might stand for. It is also important to remember that this finding concerns only the pre-program test and showed a relatively large within-group effect size of 0.57 over time.

The finding that post-program perception of financial problems predicted recidivism for program completers might be related to the fact that work with financial problems and vocational status is not included in OTO, although problem-solving is a major component of the program. This finding suggests that money and financial problems are an important issue to address within the program, for example as in the Community Reinforcement Approach (CRA; 40), where the therapist acts as a coach for job searching skills, including how to speak to persons at relevant authorities, for instance in order to restructure the participant's debts. In CRA participants are also encouraged to form job searching clubs, yielding more opportunities for exercising and practicing these skills. Whether this could be feasible in within the prison and probation setting as well as a good fit for the OTO program needs to be further explored.

Program completers who were reconvicted had a higher belief in Chance locus of control than non-recidivists, in line with previous studies (41, 42) as well as the previous finding that the Chance subscale correlates with sociopathy (43). Interestingly, only the Chance subscale was of importance in relation to re-offending, while Internal or Powerful Others loci of control were not problematic. During the cognitive restructuring parts of the OTO program, the participant's locus of control is addressed in terms of whether the participant externalizes control; it might be wise to consider addressing Chance locus of control specifically instead of working with External locus of control as a uniform concept. In terms of comparison with normative data on Chance locus of control, program completers did not differ from US student norms pre- or post-program, in line with (44) findings in a study of prisoners. However, it should be noted that the norms used might not necessarily be valid in Sweden and as a population mean. It may also be the case that those with criminal convictions need to believe in a Chance locus of control in order to motivate them to take the risks involved, but that the mean does not necessarily differ from the normal population.

### Strengths and Limitations

This study had several strengths. The evaluation of pre- and post-program test data in a national population of OTO participants between 2000 and 2008 was the first in Sweden as well as internationally. The associations with background and reconviction data give meaning to the findings, particularly in relation to the later effect study comparing OTO participants to matched controls (14). Nonetheless, the current study was limited by the lack of psychometric data and norms on the tests used, the lack of any control group, and missing data common to naturalistic studies in prison settings; an additional limitation was the lack of data on staff qualifications as well as the lack of fidelity measures for program adherence. Specific comments on the strengths and limitations of the tests used follow below.

The tests used for measures to assess progress in the program are not readily available. Only the LLOCS test was found to be previously validated, with some US norm data available but none in a Swedish population. Although each test has a clear source (see **Table 2**), the source for the PCL was not readily available. Nonetheless, the tests used in OTO appear to have good face validity. For example, the concept of locus of control is explicitly addressed in OTO in relation to problem-solving, and an association between locus of control and perceived problem-solving skills has been found in male university students (45). The Skill Survey asks about the participant's perceived skillfulness in specific areas that are worked with in OTO, and as such has merit in identifying where there have been changes. The Citizen Scale measures participants' perceptions of how they rate themselves on attitudes to laws, following rules, cooperation, and honesty, and the focus of sessions on cognitive restructuring, morals, and role rotating tasks might influence the participant's attitudes in a way that can be measured by this test. In this study, no checks were conducted for social desirability for these tests, and this is a factor that may affect how individuals report data. Since no normative studies have been conducted in a more readily comparable population, it is difficult to infer anything meaningful about the magnitude or clinical significance of any reported changes in scores. Nonetheless, the results of this study could be of use to counselors in building a personalized theory of the participant's offending. Since our findings show that some test results relate to re-offending, if norms should become available then deviations from norms for convicted and general populations might be of special interest both to counselors and participants in the assessment part of the program.

## Conclusions

The theory behind offending behavior programs in general and the OTO-program in particular, is that various aspects of psychological change mediate program outcome in terms of recidivism (46). In OTO, the psychological focus lies in the areas of problem-solving, values, motivation, social skills, and certain problem areas, and addressing these areas within the program is assumed to mediate a lower rate of recidivism. Although this study identified pro-social changes over time in the program target areas, in the absence of a control group no causal effects for the program can be ascertained. Regarding the problems associated with recidivism, mainly financial problems stand out. This aspect is not clearly linked to the theory behind the OTO program and not directly addressed within the program, but is measured pre- and post-delivery and could be addressed in tailored versions of the program. Based on the theory behind the program it was surprising that the internal-external locus of control dimension had no significance; still, our finding that Chance locus of control predicted recidivism lends some support to the idea that locus of control is meaningful to address within OTO.

The basis for the OTO-program is problem-solving and the test used to measure this aspect, the ATT, did not discriminate between recidivists and non-recidivists, and had no predictive properties. No evidence was found for the assumption that improved problem-solving ability mediated reduced recidivism. We suggest that the ATT is not the most adequate test to measure changes in problem-solving. One alternative could be the Means-Ends Problem Solving Test (MEPS), which has been used in adult populations (e.g., 47) and has shown differentiating properties for maladjustment; this test is based on the same theoretical foundation as the ATT, and was developed by the same researchers, but aims at another aspect of problem-solving, namely means-ends problem solving thinking. Another approach would be to consider the recommendations of D'Zurilla and Maydeau-Olivares (48) for using the IDEA (Inventory of Decisions, Evaluations, and Actions) as an outcome measure, or the SPSI-R (Social Problem-Solving Inventory-Revised) as a process measure of social problem solving. We do recognize, however, that one advantage of the ATT is that it is easy to administer and score and that it measures an actual skill, reducing bias, and social desirability.

The concept of psychological change leading to reduced recidivism is not new, but empirical findings are scarce on mechanisms driving such change and its translation to desistance from criminal recidivism. Much of the literature on the concept derives from theory on the Psychology of Criminal Conduct (PCC) originally described by Andrews and Bonta in several editions of their seminal book with this name (e.g., 5). A recent in-depth discussion on the PCC suggested that although dynamic risk factors have good predictive validity, the way in which psychological changes in these criminogenic needs convert to empirical outcomes has not been explored sufficiently (46). While this study focuses specifically on change in these dynamic risk factors in relation to reconviction, information on the nature of the mediating path between psychological change and reduced criminal behavior has not been part of the data set. Thus, although we identified findings that partly support the existence of a relationship between psychological changes that take place during the program and recidivism, this relationship is far from clear or unambiguous and it is important to note that no support for the theory of problem-solving deficits as related to offending was found in this study, perhaps due to the fact that the test used to measure problem-solving within OTO is not well-suited. Although we cannot draw any far-reaching conclusions based on the results of this study, they are sufficiently interesting to warrant further examination of the program with a stronger research design, particularly one that illuminates the path between in-program changes and later desistance from criminal behavior. Future studies should include tests validated with the target population and, if possible, population norms from general population and convicted samples. The problem-solving test should also include a wider range of problem solving aspects. Data collection within the prison and probation setting should prioritize high data input, possibly by using online forms centrally coordinated rather than relying on program counselors to collect data. In order to strengthen the design and allow for causal conclusions, pragmatic clinical trial design should be used (49), as well as single-case studies to closely study the path between psychological change and later behavior (50). A valuable aspect of this study could be a focus on mapping changes in emotions as addressed in relation to childhood schemas (46). Finally, from a practical point of view, it could be expedient to evaluate age-appropriate interventions in comparison to OTO participation, to investigate whether younger people might show greater retention and success in reducing criminal behaviors compared with OTO in its current version. Practical interventions to reduce financial problems at the end of the program could also be added to OTO as an additional session focusing, for example, on job searching clubs and debt restructuring. The OTO program has been shown successful in reducing recidivism. It has long been offered in several criminal justice services internationally. This well-founded program could be further improved if the recommendations from this study are followed.

## Data Availability Statement

The raw data supporting the conclusions of this manuscript will be made available by the authors to any qualified researcher.

## Author Contributions

Authors PP and AB conceived the study design, and PM and MG carried out the analyses within their thesis work for the MSc degree in Clinical Psychology, under AB's supervision. This manuscript, based on the thesis originally written by PM and MG, was revised and edited by AB with input from PP, PM, and MG. All authors approved the final manuscript and are accountable for its contents.

## Funding

AB was funded for salary by Swedish Research Council grant nr K2012-61-P-22131-01-6. She participated in this work within the frame of the Swedish program grant "Responding to and Reducing Gambling Problems—Studies in Help-seeking, Measurement, Comorbidity and Policy Impacts" (REGAPS), financed by the Swedish Research Council for Health, Working Life and Welfare (Forte), grant number 2016-07091. Other authors were funded within their employment.

## Conflict of Interest

PP is the author of the One-to-One program, and trained criminal justice staff (1990 – 2003) to deliver it in the UK, Sweden, and Norway. He has no current involvement with the program in any of those locations. 

The remaining authors declare that the research was conducted in the absence of any commercial or financial relationships that could be construed as a potential conflict of interest.

## Footnotes

1 The number of previous offenses could not be included because there were only 144 valid cases for this variable.
